# Toxic Peptide From *Palythoa caribaeorum* Acting on the TRPV1 Channel Prevents Pentylenetetrazol-Induced Epilepsy in Zebrafish Larvae

**DOI:** 10.3389/fphar.2021.763089

**Published:** 2021-12-01

**Authors:** Xiufen Wang, Qiwen Liao, Hanbin Chen, Guiyi Gong, Shirley Weng In Siu, Qian Chen, Hiotong Kam, Carolina Oi Lam Ung, Kwok-Kuen Cheung, Gandhi Rádis-Baptista, Clarence Tsun Ting Wong, Simon Ming-Yuen Lee

**Affiliations:** ^1^ State Key Laboratory of Quality Research in Chinese Medicine and Institute of Chinese Medical Sciences, University of Macau, Macau, China; ^2^ School of Life and Health Sciences, The Chinese University of Hong Kong, Shenzhen, China; ^3^ Department of Computer and Information Science, Faculty of Science and Technology, University of Macau, Macau, China; ^4^ Department of Rehabilitation Sciences, The Hong Kong Polytechnic University, Hong Kong, China; ^5^ Laboratory of Biochemistry and Biotechnology, Institute for Marine Sciences, Federal University of Ceará, Fortaleza, Brazil; ^6^ Department of Applied Biology and Chemical Technology, The Hong Kong Polytechnic University, Hong Kong, China

**Keywords:** zoantharian, PcActx peptide, transcriptomics analysis, TRPV1 channel, anti-epilepsy, zebrafish

## Abstract

PcActx peptide, identified from the transcriptome of zoantharian *Palythoa caribaeorum,* was clustered into the phylogeny of analgesic polypeptides from sea anemone *Heteractis crispa* (known as APHC peptides). APHC peptides were considered as inhibitors of transient receptor potential cation channel subfamily V member 1 (TRPV1). TRPV1 is a calcium-permeable channel expressed in epileptic brain areas, serving as a potential target for preventing epileptic seizures. Through *in silico* and *in vitro* analysis, PcActx peptide was shown to be a potential TRPV1 channel blocker. *In vivo* studies showed that the linear and oxidized PcActx peptides caused concentration-dependent increases in mortality of zebrafish larvae. However, monotreatment with PcActx peptides below the maximum tolerated doses (MTD) did not affect locomotor behavior. Moreover, PcActx peptides (both linear and oxidized forms) could effectively reverse pentylenetetrazol (PTZ)-induced seizure-related behavior in zebrafish larvae and prevent overexpression of *c-fos* and *npas4a* at the mRNA level. The excessive production of ROS induced by PTZ was markedly attenuated by both linear and oxidized PcActx peptides. It was also verified that the oxidized PcActx peptide was more effective than the linear one. In particular, oxidized PcActx peptide notably modulated the mRNA expression of genes involved in calcium signaling and γ-aminobutyric acid (GABA)ergic-glutamatergic signaling, including *calb1, calb2*, *gabra1*, *grm1*, *gria1b*, *grin2b, gat1*, *slc1a2b, gad1b,* and *glsa*. Taken together, PcActx peptide, as a novel neuroactive peptide, exhibits prominent anti-epileptic activity, probably through modulating calcium signaling and GABAergic-glutamatergic signaling, and is a promising candidate for epilepsy management.

## Introduction

The Phylum Cnidaria, containing almost 13,000 species, is classified into five main classes: Anthozoa, Cubozoa, Hydrozoa, Scyphozoa, and Staurozoa (Kayal et al., 2013). The classes Myxozoa and Polypodiozoa are also recognized as cnidarians (Jimenez-Guri et al., 2007). Cnidocytes are specific to cnidarians, which convey venomous compounds to other organisms for defense or predation (Jouiaei et al., 2015). Phylum Cnidaria represents a large number of bioactive peptide toxins that could contribute to the development of drug therapies, owing to their selective and specific interaction with diverse kinds of ion channels, including voltage-gated potassium (K_V_), calcium (Ca_V_) and sodium (Na_V_) channels, the acid-sensing ion (ASIC) channel, and the TRPV1 channel (Mouhat et al., 2004).

Of particular interest is Anthozoa which is currently categorized into 11 orders and almost 7,000 species (such as sea anemones, corals and zoanthids), comprising about 65% of all known cnidarian species. Most sources of the bioactive peptides of Anthozoa were identified from sea anemones. For example, Stichodactyla toxin (ShK) and its analogs, which were found in the sea anemone *Stichodactyla helianthus*, could specifically bind to the K_V_1.3 ion channel, potentially serving as effective therapies for autoimmune diseases (Castaneda et al., 1995; Norton et al., 2015). An analog of ShK, named ShK-186 or dalazatide, is currently in human clinical trials (Tarcha et al., 2017). Another example is the peptide APETx2, derived from sea anemone *Anthopleura elegantissima* (Diochot et al., 2004). A previous report showed that it exerted analgesic effects in an inflammatory pain model through inhibition of the ASIC3 channel (Karczewski et al., 2010). Recently, understanding of the venom peptide components in anthozoan species other than sea anemones has allowed novel bioactive peptides with important pharmacological effects to be discovered. As a zoantharian, a subclass of Hexacorallia, *Palythoa caribaeorum* is in the sister clade of sea anemone (Kayal et al., 2017). Its mucus, traditionally used in folk medicine in northeastern Brazilian coastal regions, has properties that make it useful as an anesthetic, analgesic and anti-inflammatory agent, along with healing properties in the treatment of topical wounds (de Andrade Melo et al., 2012). It was reported that the venom of *P. caribaeorum* delays inactivation of the Na_V_1.7 channel and inhibits Ca_V_2.2 channel and K_V_ (I_A_ and I_DR_ currents) channels on superior cervical ganglion (SCG) neurons (Lazcano-Perez et al., 2016). In our previous studies, two peptides from *P. caribaeorum*, namely PcShK and PcKuz, were shown to act on the K_V_ ion channel and displayed neuroprotective and cardioprotective effects *in vitro* and *in vivo* (Liao et al., 2018a; Liao et al., 2018b).

APHC peptides from sea anemone *Heteractis crispa* were firstly reported as peptide antagonists of the TRPV1 channel (Andreev et al., 2008). APHC3 displayed the most potent inhibitory effect (71%) on capsaicin-induced TRPV1 activation (Nikolaev et al., 2017). *In vivo* studies showed that APHC peptides produced significant analgesic effects in different pain models (Andreev et al., 2008; Andreev et al., 2013). In addition, HCRG21, obtained from the sea anemone *H. crispa*, shares high structural homology with APHC peptides and fully inhibits the TRPV1 channel (Monastyrnaya et al., 2016).

The TRPV1 channel belongs to subfamily vanilloid of the transient receptor potential (TRP) cation channel (Montell et al., 2002). It is a tetrameric architecture, the subunits of which exhibit four-fold symmetry around a central ion-conducting pathway (Liao et al., 2013). Each subunit has six transmembrane domains (S1-S6), a hydrophobic pore located between S5 and S6, and cytosolic C- and N-terminal tails (Liao et al., 2013). The transmembrane domains S5, S6 and their linker form the central ion permeation pore, while S1-S4 and the cytosolic N- and C-terminals are considered to regulate channel gating (Vay et al., 2012). In addition, the TRPV1 channel allows Ca^2+^ to pass through the cell membrane via non-selectively hydrophobic pores (Ramsey et al., 2006). The TRPV1 channel was discovered in primary sensory neurons and non-neuronal cells (Caterina et al., 1997; Toth et al., 2005; Jeffry et al., 2009), and was activated by noxious heat (≥43°C) and inflammatory substances, such as N-arachidonoylethanolamide, as well as protons (low pH), which contribute to pain hypersensitivity (Liao et al., 2013). It can also be activated by capsaicin, oxidative stress, hydrogen peroxide, nitric oxide, and oxidized linoleic acid (Naziroglu, 2015).

Moreover, the TRPV1 channel has been confirmed to be expressed in dentate gyrus and the CA1 area of the hippocampus, which acts as an important site for epilepsy induction (Roberts et al., 2004; Toth et al., 2005). Epilepsy is pathologically characterized by an imbalance between excitatory glutamate transmission and inhibitory GABA impulses, and abnormal activation of ion channels (Na^+^, K^+^ and Ca^2+^) (Jefferys, 2010). Reports showed that TRPV1 channel activation enhanced glutamate release and glutaminergic signaling (Gerdeman and Lovinger, 2001; Shoudai et al., 2010; Fawley et al., 2014), decreased GABA release (Gonzalez-Aparicio and Moratalla, 2014; von Ruden et al., 2015), and induced Ca^2+^ accumulation (Naziroglu et al., 2014), which were confirmed to be responsible for synaptic efficacy and epilepsy. Overload of cytosolic Ca^2+^ causes severe mitochondrial dysfunction, and thereby increases the generation of ROS and release of apoptosis-related factors (like caspase 3 and 9) (Naziroglu et al., 2014; Ovey and Naziroglu, 2015). Meanwhile, ROS formation is recognized to further aggravate epileptic seizure via TRPV1 activation (Naziroglu, 2015). However, the TRPV1 channel blockers, capsazepine and 5′-iodoresiniferatoxin (IRTX), exhibited protective effects against epilepsy-induced Ca^2+^ influx and apoptosis via the TRPV1 channel in hippocampal and dorsal root ganglion (DRG) neurons (Ghazizadeh and Naziroglu, 2014; Naziroglu et al., 2015; Naziroglu and Ovey, 2015). The TRPV1 channel agonist capsaicin significantly increased the spontaneous excitatory synaptic transmission induced by glutamate, while it was reversed by treatment with both capsazepine and IRTX, indicating their modulation of glutamatergic systems (Starowicz et al., 2007; Bhaskaran and Smith, 2010). Taken together, a reduction of calcium accumulation and regulation of glutaminergic systems through inhibition of the TRPV1 channel can be exploited to induce neuronal protective effects against epileptic seizure.

We previously performed deep RNA-Seq of the whole transcriptome of *P. caribaeorum* which was deposited at DDBJ/EMBL/GenBank under the accession number of GESO00000000 associated with the BioProject PRJNA320984 (Liao et al., 2018b). A series of peptide precursors containing a Kunitz domain were identified, and some of these peptides are homologous with APHC peptides, which are defined as PcActx peptide in the present study. Since APHC peptides have been reported and confirmed as TRPV1 inhibitors, here we aimed to investigate the structure, bioactivity and mechanism of action of PcActx peptide identified from transcriptomics analysis of *P. caribaeorum*.

## Materials and Methods

### Primary Structure Analysis

The peptide sequences, which the candidate peptide might be homologous, were obtained from the UniProtKB database. Phylogenetic analysis was conducted using the program MEGA, version 6 based on the maximum-likelihood method (Tamura et al., 2013), followed by sequence editing and alignment using the MUSCLE algorithm (Edgar, 2004a; b). The bootstrap method was used to validate the reliability of the phylogenetic tree.

### Structural Modeling and Molecular Dynamics Simulation

Structures of APHC1 peptide (UniProt ID: B2G331), PcActx peptide and PcActx peptide dimer were homology modeled using the SWISS-MODEL server (Biasini et al., 2014). The GROMACS 5.1 simulation software (Pronk et al., 2013) was used to perform MD simulations with the CHARMM27 all-atom force field. The modeled peptide was firstly solvated with TIP3P water before subjected to equilibration in 300 K for 10 ns, with energy minimization in 5 × 10^7^ steps. The production step is run for 100 ns, with a 2 fs timestep and van der Waals interaction cutoff of 1.2 nm. The thresholds 300 thermostat and 1.0 barostat were used to generate the NPT ensemble. Particle-meshed Ewald (PME) was carried out for long-range electrostatics. Structural alignment was performed to compare the equilibrated structures to the analgesic peptide APHC1 from sea anemone, which was similar to the PcActx peptide. Molecular visualization was achieved using the PyMOL program (version 1.8, Schrödinger, LLC).

### Peptide Synthesis and Oxidative Folding

The linear PcActx peptide was synthesized by standard Fmoc solid phase chemistry (GL Biochem, Shanghai, China). All the Cys on the peptide used Cys (Trt) as protecting group. After the synthesis, complete cleavage and deprotection were performed using trifluoroacetic acid in water to remove all the protecting groups on the peptide. The precipitated peptide was obtained by the addition of chilled ether. The crude peptide was purified by RP-HPLC (Agilent Technologies, Santa Clara, CA, United States) using Kromasil 100-5 C18 column (5 μm, 4.6 mm × 150 mm) at a flow rate of 1 ml min^−1^. The mixture of 0.1% trifluoroacetic in acetonitrile (mobile phase A) and water (mobile phase B) was used with the following gradient: 0–25 min, 25–50% A; 25–25.1 min, 50–100% A; 25.1–30 min, 100% A. The peptide was freeze-dried and retested by RP-HPLC to ensure its purity which was higher than 90% with the presence of one single peak. ESI-MS (Agilent Technologies) analysis was performed to confirm the molecular weight (MW) of the linear PcActx peptide. Operating conditions optimized for the detection of reaction mixture were the followings: Gas temperature: 300°C, Drying gas: 8 L min^−1^, Nebulizer: 35 psig, Sheath gas temperature: 350°C, Sheath gas flow: 11 L min^−1^, VCap: 3,500 V, Nozzle voltage: 1,000 V.

Oxidative folding was performed to achieve the oxidized PcActx peptide after synthesizing the linear form (Liao et al., 2019). Linear PcActx peptide was solubilized in sodium carbonate buffer (pH 8.0) at the final concentration of 100 μM, in the presence of reduced and oxidized glutathione of ratio 5:1 for 48 h at room temperature. Nitrogen gas was blown into the solution to protect the peptide against the air oxidation during the reaction. The oxidative reaction was terminated by acidification. Then, the oxidized peptide was purified and retested by RP-HPLC (Waters Corporation, Milford, MA, United States) using XBridge BEH300 C18 column (5 μm, 4.6 mm × 150 mm) at a flow rate of 1 ml min^−1^ with the gradient of 10–80% acetonitrile in 40 min. The ESI-MS analysis was performed to confirm the MW of the oxidized PcActx peptide.

Then the oxidized peptide was incubated in dd-H_2_O for 48 h at 1 mM at room temperature. The mass identification of monomeric and dimeric peptides was conducted by ESI-MS. The linear and oxidized PcActx peptides were dissolved in dd-H_2_O as a stock solution (10 mM) and stored at - 20 °C for bioactive assay.

### Peptide-Protein Docking Analysis

The atomic coordinates of the TRPV1 channel were homology-modeled using the SWISS-MODEL server. The interactions between the PcActx peptide and TRPV1 channel and the interactions between the APHC1 peptide and TRPV1 channel were modeled using ZDOCK, which is a fast Fourier transform (FFT)-based, initial-stage rigid-body molecular docking algorithm (Pierce et al., 2011). The PyMOL program was used for molecular visualization (Humphrey et al., 1996).

### Cell Culture and Fluorescent Calcium Measurement

The HEK293 cells (ATCC, Manassas, VA, United States) were maintained in DMEM medium (Gibco, Carlsbad, CA, United States) containing 10% (v/v) fetal bovine serum (FBS, Gibco), and 1% (v/v) penicillin-streptomycin (PS, Gibco), and incubated in a humidified atmosphere of 5% CO_2_ at 37°C. HEK293 cells stably expressing human TRPV1 (HEK293-hTRPV1 cell) were generated using Lipo8000™ Transfection Reagent (Beyotime Company, Shanghai, China) according to manufacturer’s protocol. Briefly, HEK293 cells were transfected with 2.5 μg of plasmid DNA encoding a human TRPV1 protein (NM_018727.5, IGE Biotechnology, Guangzhou, China). Cells were seeded on the 12-well plate and cultured overnight at 37°C. After pretreatment with PcActx peptide (1 and 0.5 μM) or capsazepine (0.25 μM, TRPV1 antagonist, MCE, NJ, United States) for 4 h, the cells were stained with 2 μM Fluo-4/AM (Invitrogen, San Diego, CA, United States) in dark at 37°C for 30 min in HEPES buffer (NaCl 140 mM, KCl 5 mM, HEPES 10 mM, MgCl_2_ 2 mM, CaCl_2_ 2 mM, glucose 10 mM, pH 7.4) and then washed with HEPES buffer three times. Subsequently, the cells were challenge with 10 nM capsaicin (TRPV1 agonist, Sigma-Aldrich, St. Louis, MO. United States) and the calcium levels were immediately detected using the cellSens imaging system of an IX73 microscope (Olympus Co., Tokyo, Japan). The excitation wavelength used was 494 nm and emission wavelength was measured at 516 nm.

### Zebrafish Maintenance

AB wild-type zebrafish (*Danio rerio*) were manipulated under standard environmental conditions with a controlled temperature (28.5°C) and light (14 h/10 h light/dark cycle) (Westerfield, 2000). The embryos were collected after natural pairwise mating (3–12 months). Then, the embryos and larvae were raised in an incubator system maintained at 28.5°C in embryo medium.

### Survival Rate Determination

Zebrafish larvae at 5-day post-fertilization (5-dpf) were exposed to different concentrations (linear form: 0–80 μM, oxidized form: 0–40 μM) of PcActx peptides for 24 h. The mortality of zebrafish was evaluated by determining the presence or absence of heartbeat. The survival rates, LC_50_ values and MTD values were calculated.

### Locomotion Behavioral Test

#### Locomotion Assay on Normal Zebrafish

Zebrafish larvae (5-dpf) were exposed to PcActx peptides at different concentrations below the MTD (linear form: 5–20 μM, oxidized form: 2.5–10 μM) for 24 h. Then, they were transferred into a 96-well plate (1 fish/well). Zebrafish locomotion was measured for 60 min using a zebrafish tracking system (Viewpoint Life Sciences, Montreal, Canada). Six sessions (10 min each) of locomotion were recorded for each zebrafish larva. The total distance travelled by each zebrafish larva was simultaneously calculated for each recording session.

#### Locomotion Assay of PTZ-Induced Epileptic Zebrafish Model

Zebrafish larvae (5-dpf) received pretreatment with PcActx peptides at different concentrations for 24 h. Then, larvae were transferred to a 96-well plate (1 fish/well) and treated with 3.3 mM PTZ (Sigma-Aldrich) to induced epileptic seizure. Zebrafish locomotion recording was quickly initiated, and locomotion was measured for 30 min using a zebrafish tracking system (Viewpoint Life Sciences). Six sessions (5 min each) were recorded for each zebrafish larva. The total distance travelled, total distance travelled at high velocity (> 20 mm/s) and total duration of high velocity travel of each zebrafish were simultaneously calculated across sessions. The distance travelled at high velocity (>20 mm/s) was used to represent “fast” activity based on previously established protocols, which is rarely exceeded by control larvae (<5% of control activity) (Ellis et al., 2012).

### Quantitative Real-Time PCR

Zebrafish larvae (5 dpf) were treated with PcActx peptides for 24 h prior to treatment of PTZ, which proceeded for an additional 30 min. Total RNA pools of zebrafish larvae were extracted from each group using TRIzol reagent (Life Technologies, Carlsbad, CA, United States) according to the standard protocol. cDNA was reverse-transcribed from isolated RNA using the Transcriptor First Strand cDNA Synthesis Kit (Roche Applied Science, Penzberg, Germany) following the manufacturer’s protocol. RT-PCR was performed by using SYBR^®^ Premix Ex Taq™ II (TaKaRa, Shiga, Japan) on the Real-Time PCR System (Agilent Technologies) according to the manufacturer’s instructions. The mRNA expression was normalized to *ef1a*. The primer sequences used are listed in Supplementary Table S1.

### ROS Detection

The concentrations of ROS were estimated using the fluorescent probe, CM-H_2_DCFDA (Sigma-Aldrich). After treatment with different PcActx peptides for 24 h, zebrafish larvae were treated with PTZ (10 mM) and CM-H_2_DCFA (10 μM) for 1.5 h at 28.5°C in the dark. Excess CM-H_2_DCFA was subsequently washed out by embryo medium. Zebrafish larvae were observed by a Disk Scanning Unit (DSU) Confocal Imaging System (Olympus Co.). The microscopic images of zebrafish were obtained in FITC mode. The integrated intensity of ROS was quantified using ImageJ (NIH, Bethesda, MD, United States).

### Statistical Analysis

Statistical analyses were performed using GraphPad Prism software (version 8.0; GraphPad Software, Inc., San Diego, CA, United States). Data are expressed as means ± standard deviation (SD), and statistical significance was analyzed by one-way ANOVA followed by Dunnett’s test for multiple comparisons. Differences between means were considered statistically significant when *p* < 0.05.

## Results

### PcActx Peptide Sequence Clustering by APHC Peptide Phylogeny

**FIGURE 1:**
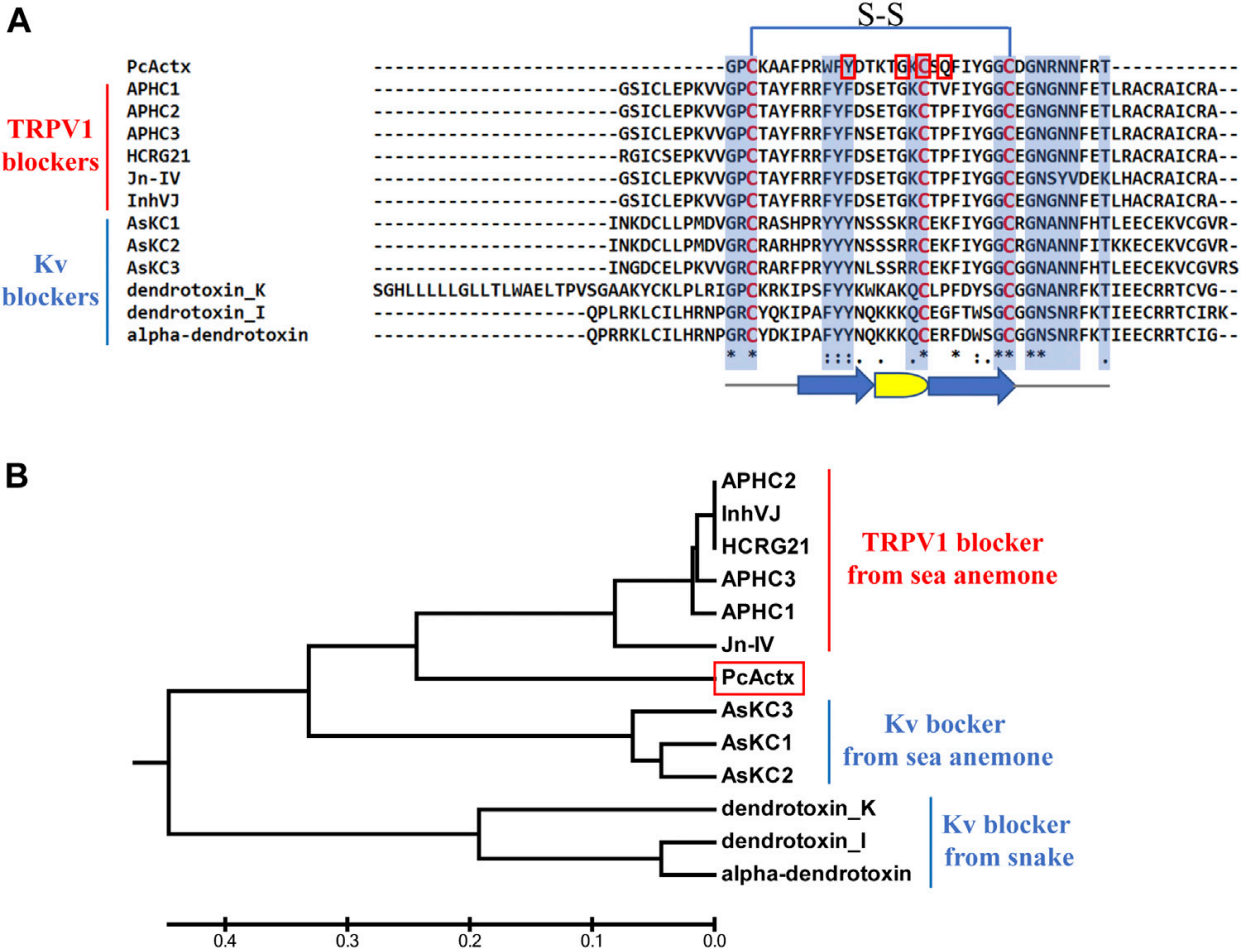
Multiple sequence alignment and phylogenetic analysis of PcActx peptide from *P. caribaeorum.* **(A)**. Multiple sequence alignment of PcActx peptide and TRPV1 blockers originating from sea anemone, as well as Kv blockers originating from sea anemone and snake. Residues highlighted in blue are the key sites interacting with ion channels. Residues highlighted in red are the interactive sites of PcActx peptides binding to the TRPV1 channel. The arrow symbols represent β-sheet, and the semicircle represents β-turn. **(B)**. Phylogenetic tree of PcActx peptide.

**FIGURE 2:**
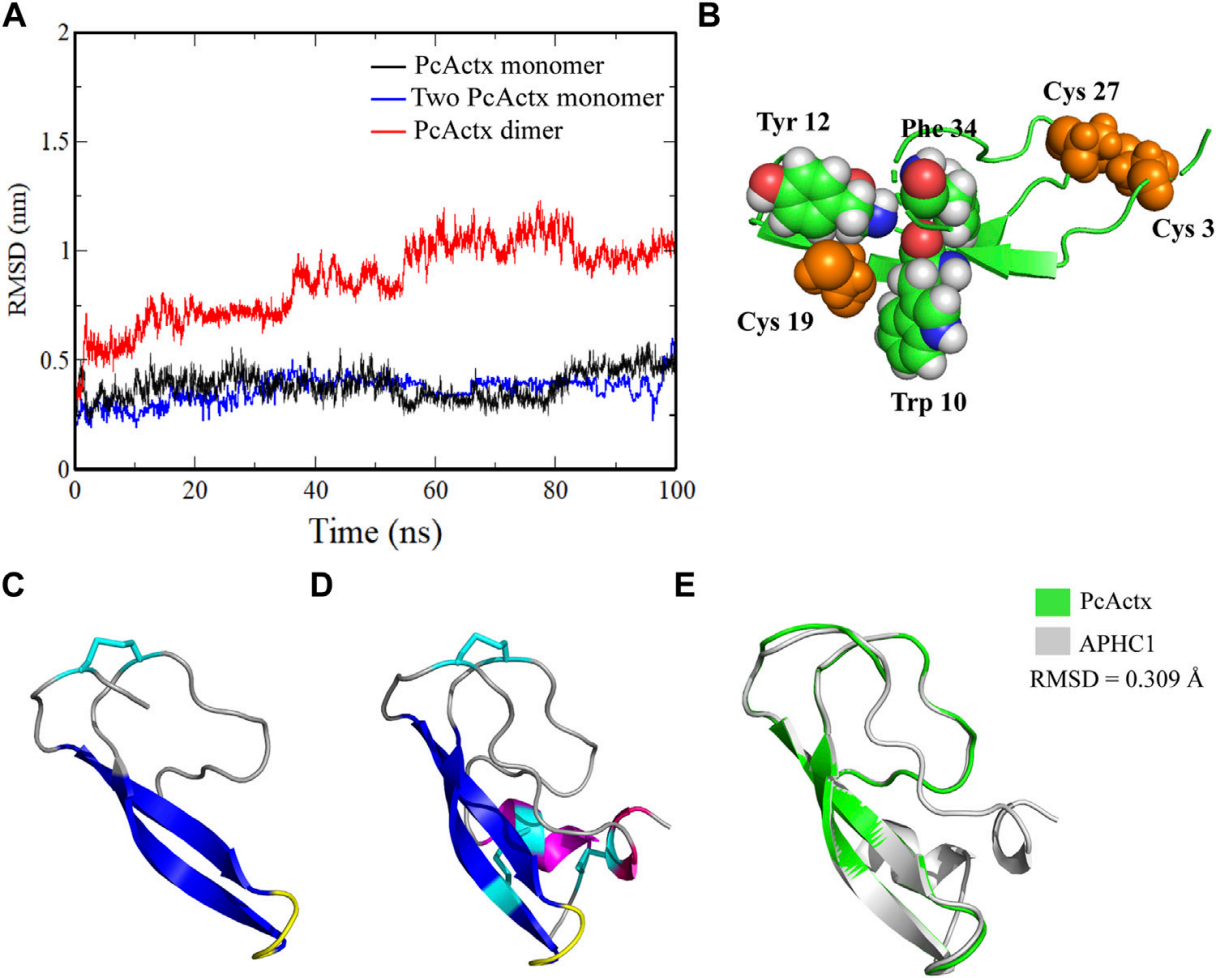
MD simulation and structure comparison. **(A)**. MD simulation of PcActx peptide monomer and dimmer. **(B)**. Spatial structure of Cys 19 of PcActx peptide. Cys residues are shown as spheres in orange. Trp10, Tyr 12 and Phe 34 residues are shown as spheres in green. Other molecules are shown as cartoons in green. **(C,D).** Homology model structure of PcActx **(C)** and APHC1 **(D)** peptide. Molecules are shown as cartoons and colored based on the secondary structure: α-helix in purple, β-sheet in blue, β-turn in yellow, coil in gray, and disulfide bond in cyan. **(E).** Structure overlap of PcActx and APHC1. The structure of PcActx peptide is shown in green, and the structure of APHC1 peptide is shown in gray.

The net charge values, p*I* values, MWs, and sequences of PcActx peptide are shown in Table 1. Multiple sequence alignment and phylogenetic analysis were employed to establish the structural relationships of PcActx peptide. As shown in the phylogenetic tree (Figure 1B), PcActx peptide was phylogenetically related to APHC and HCRG21 peptides isolated from sea anemone. Multiple sequence alignment analysis (Figure 1A) also revealed that PcActx peptide was homologous with APHC peptides. Moreover, the equilibrated PcActx structure well superposed spatially to APHC1 peptide, as shown in Figure 2E, giving a root-mean squared deviation (RMSD) value of 0.309 Å. However, APHC peptides contained six cysteine residues and formed three disulfide bridges (Andreev et al., 2008; Kozlov et al., 2009) while PcActx peptide had three cysteine residues and formed one disulfide bond (Cys 3-Cys 27, Figure 1A) with one free cysteine residue (Cys 19).

**TABLE 1:** Primary sequences and physicochemical characteristics of the PcActx peptides.

Peptides	Primary sequence	MW (monoisotopic mass, Da)	p*I*	Net charge (z)
Linear PcActx	GPCKAAFPRWFYDTKTGKCSQFIYGGCDGNRNNFRT	4104.89	9.36	3.9
Oxidized PcActx	GPC_3_KAAFPRWFYDTKTGKCSQFIYGGC_27_DGNRNNFRT	4102.53	9.36	3.9

### PcActx Peptide Functions as a Monomer Rather Than a Dimer

Since there is one single free cysteine in each PcActx peptide sequence, it is uncertain if the free cysteine residues may bound to each other to cause formation of dimer. The possibility of dimer formation was firstly determined by computational prediction. MD simulation was conducted to determine the stabilization of monomer and dimer forms of PcActx peptide. The RMSD values of PcActx peptide monomer reached a plateau at approximately 0.3 nm after 100 ns simulation (Figure 2A in black), indicating that the modeled PcActx peptide monomer is very stable. Similar result was also observed in the system of two PcActx peptide monomers (Figure 2A in blue). However, the dimer, formed by connection with an inter-chain disulfide bond at Cys 19 residues of the two peptides, significantly increased the RMSD values at about 1 nm (Figure 2A in red). One of the peptide chains in the dimer disrupted during the MD simulation, indicating the instability of the dimer structure. In addition, by looking at the structure (Figure 2B), the Cys 19 was frequently attracted by the neighbouring aromatic residues (Trp10, Tyr 12 and Phe 34), which was unfavorable to occurrence of dimerization in the Cys 19 residue.

**FIGURE 3:**
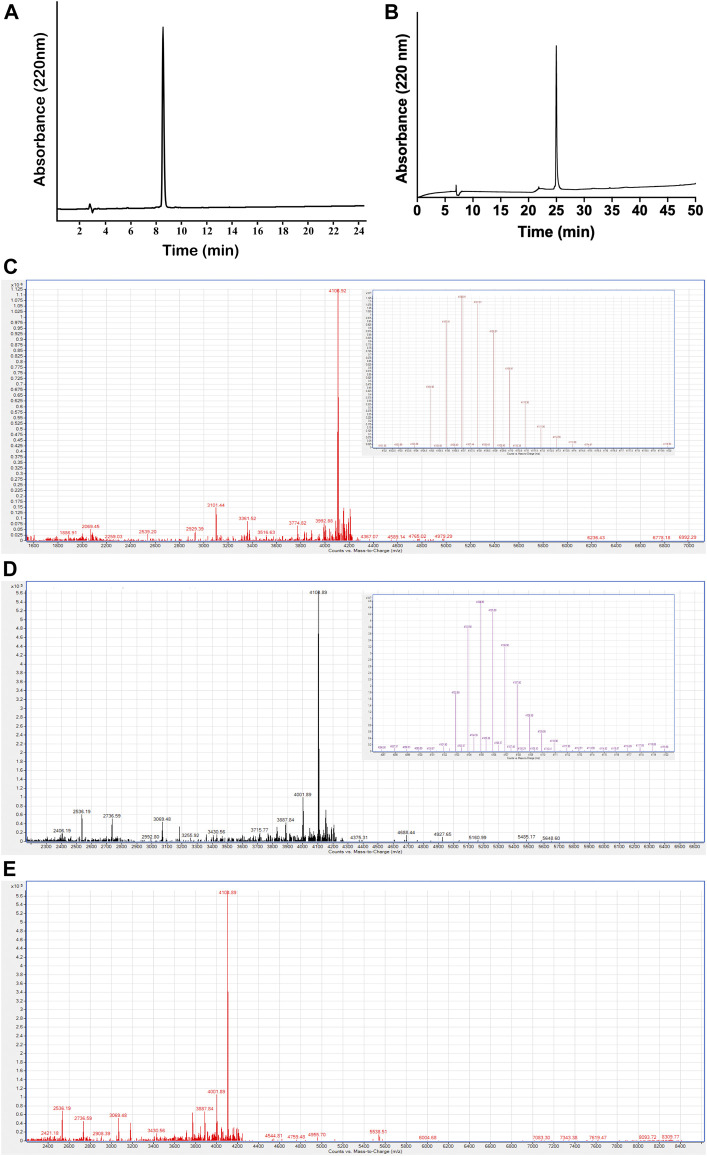
Purification and characterization of PcActx peptides. **(A)**. Analytical RP-HPLC chromatograph for the final purified linear PcActx peptide with absorbance at 220 nm with purity ≥90%. **(B)**. Analytical RP-HPLC chromatograph for the final purified oxidized PcActx peptide with absorbance at 220 nm with purity ≥90%. **(C)**. ESI-MS analysis of linear PcActx peptide. Anal. calcd for C_182_H_265_N_53_O_51_S_3_ [M + H]^+^ 4104.89 Da (monoisotopic mass), found [M + H]^+^ 4104.90 Da. **(D)**. ESI-MS analysis of oxidized PcActx peptide. Anal. calcd for C_182_H_263_N_53_O_51_S_3_ [M + H]^+^ 4102.87 Da (monoisotopic mass), found [M + H]^+^ 4102.88 Da. **(E)**. ESI-MS analysis of oxidized PcActx peptide incubated in dd-H_2_O for 48 h.

Moreover, in addition to computational prediction, the feasibility of dimer formation of the PcActx peptide was further evaluated by a chemical experiment. Linear and oxidized PcActx peptides, the purity grade (>90%) and MWs (monoisotopic mass, 4104.89 and 4102.87 Da, respectively) were produced and analyzed by RP-HPLC and MS (Figures 3A–D). Then the oxidized peptide was incubated in dd-H_2_O for 48 h, for checking if it might form dimer under experimental condition. After incubation for 48 h, no dimer mass was found in the solution as analyzed by ESI-MS (Figure 3E). Therefore, both computational and chemical experiments also suggest that the PcActx peptide tends to form a monomer rather than a dimer.

### Computational Prediction and Biological Validation of PcActx Peptide as a Ligand of the TRPV1 Channel

**FIGURE 4:**
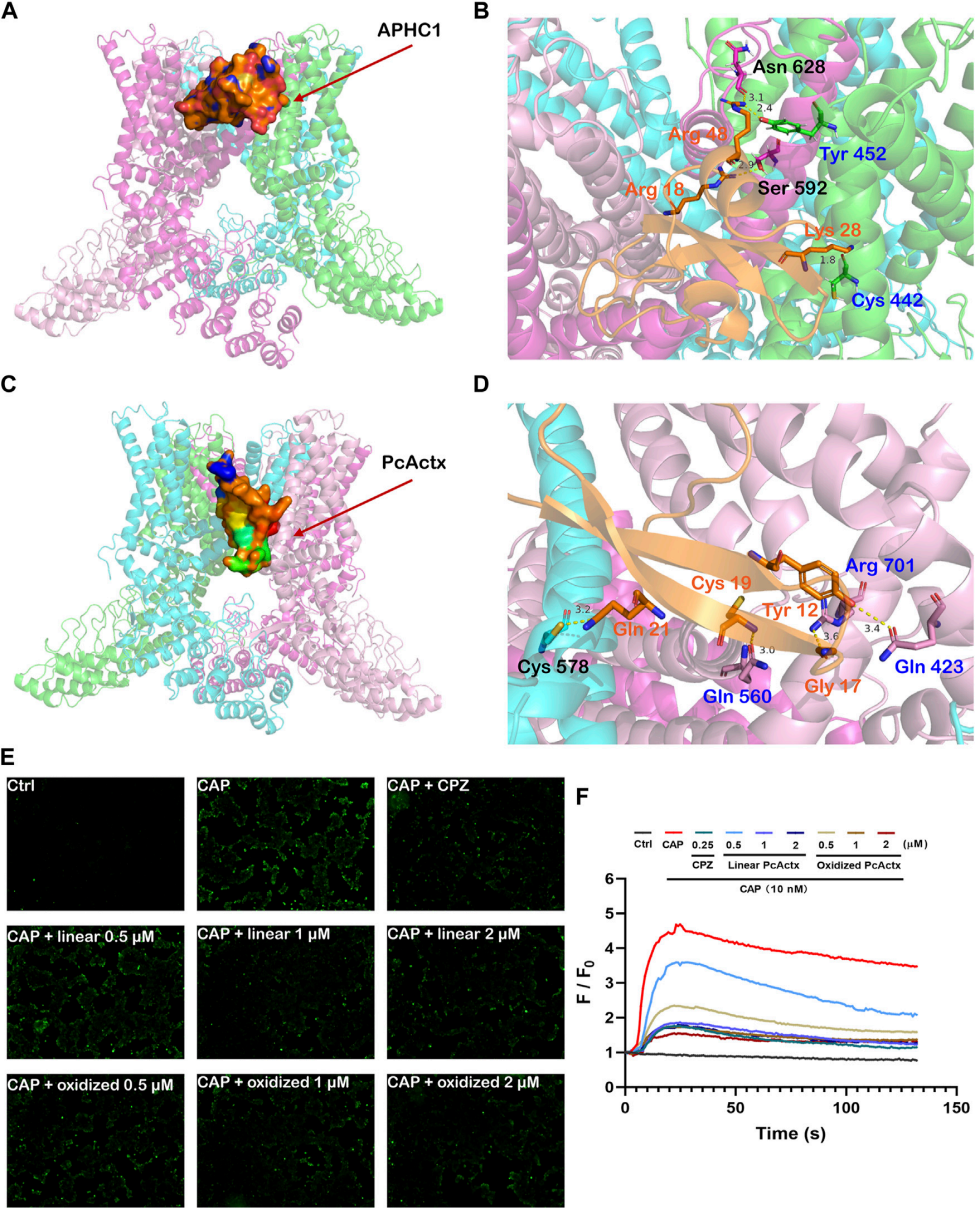
Peptide-protein docking analysis and fluorescent calcium measurement. **(A)**. Side view of protein-peptide docking of the APHC1 peptide binding to the TRPV1 channel. The peptide is shown as a ribbon diagram colored in orange; the protein is shown as a cartoon. **(B)**. Interface residues between APHC1 peptide and the TRPV1 channel. APHC1 peptide is shown in orange cartoon. In α chain, the hydrogen bonds are formed by Arg 18 and Arg 48 of APHC1 peptide with residue Ser 592 and Asn 628, respectively. In β chain, the hydrogen bonds are formed by Lys 28 and Arg 48 of APHC1 peptide with residue Cys 442 and Try 453, respectively. **(C)**. Side view of protein-peptide docking of the PcActx peptide binding to the TRPV1 channel. The peptide is shown as a ribbon diagram colored in orange; the protein is shown as a cartoon. **(D)**. Interface residues between PcActx peptide and the TRPV1 channel. PcActx peptide is shown in orange cartoon. The hydrogen bonds are formed by Tyr12, Gly17, Cys19, and Gln21 of PcActx peptide with residue Gln423, Arg701, Gln560 in γ chain, and Cys578 in δ chain of TRPV1, respectively. **(E)**. Representative images of intracellular calcium concentration of HEK293-hTRPV1 cell (CAP: capsaicin; CPZ: capsazepine). **(F)**. Representative time-dependent response of Ca^2+^ fluorescence intensity in each group. Ca^2+^ responses were measured as changes in fluorescence intensity of the representative average plots (n = 5) before (F_0_) and after capsaicin addition (F). **(E,F)** graphs represent a single representative experiment from a total of three independent experiments.

Since PcActx peptide was phylogenetically close to APHC peptides, which were reported as TRPV1 inhibitors, we hypothesized that PcActx might also bind to the TRPV1 channel. The docking analysis of APHC1 peptide and PcActx peptide to TRPV1 channel were performed and compared. As depicted in Figures 4A–D, it seems that both APHC1 and PcActx could bind with two adjacent subunits of TRPV1 channel. As a positive control, APHC1 peptide interacted with S1, S5 and S5-P-S6 domains of the TRPV1 channel. Residues Arg 18 and Arg 48 of APHC1 peptide interacted with residues Ser 592 and Asn 628 in α chain with distances of 2.9 Å and 3.1 Å, respectively. Residues Lys 28 and Arg 48 of APHC1 peptide interacted with residues Cys 442 and Try 453 in β chain with distances of 1.8 Å and 2.4 Å, respectively. Meanwhile, PcActx peptide could interact with the pre-S1 helix, S4-S5 linker and TRP domain of the TRPV1 channel via four hydrogen bonds (Figures 4C,D). Residues Tyr 12, Gly 17, Cys 19 and Gln 21 were the interactive sites of PcActx peptide. In γ chain of the TRPV1 channel, Tyr12, Gly17 and Cys19 of the PcActx peptide interacted with Gln 423, Arg 701, and Gln 560 with distances of 3.6 Å, 3.4 Å and 3.0 Å, respectively. In δ chain of the TRPV1 channel, Gln 21 of the PcActx peptide interacted with Cys 578 with a distance of 3.2 Å. Therefore, both APHC1 and PcActx could potentially bind with two adjacent subunits of TRPV1 channel, but they interacted with different structural domains, respectively.

Moreover, the effect of PcActx peptide on TRPV1 channel mediated calcium influx in cultured cell was determined to confirm the above computational prediction. As shown in Figures 4E,F, 10 nM capsaicin, the TRPV1 agonist, can obviously induce the calcium influx in HEK293-hTRPV1 cell at which HEK293 was engineered to express recombinant hTRPV1 protein. However, no obvious calcium response was observed when the non-expressing TRPV1-HEK293 cell were treated with capsaicin at dosages ranging from 10–2 mM (Supplementary Figure S1), indicating that capsaicin significantly induced TRPV1-dependent calcium influx. Similar to the TRPV1 channel antagonist capsazepine, linear PcActx pepetide could markedly abated the calcium accumulation at the concentration of 1 and 2 μM. Furthermore, treatment with 0.5 to 2 μM oxidized PcActx peptide also efficaciously inhibited the calcium response evoked by capsaicin (Figures 4E,F). Therefore, we believe that the PcActx peptide suppressed the capsaicin-induced calcium influx through the TRPV1 channel inactivation.

### Survival Rates of Zebrafish Larvae After Exposure to PcActx Peptides

**FIGURE 5:**
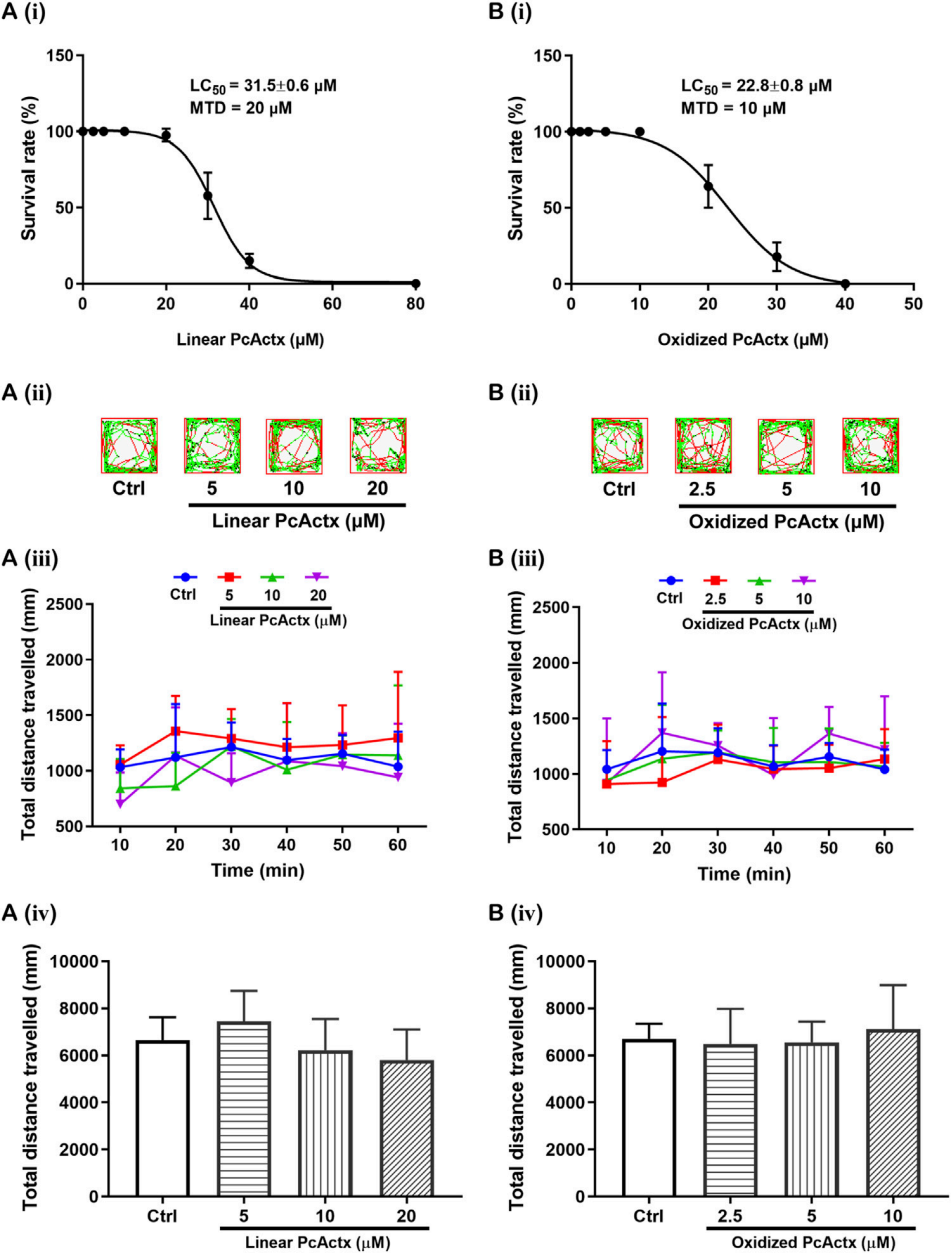
Survival and behavioral toxicity of zebrafish larvae after 24 h exposure to PcActx peptides. **(A)** Linear PcActx peptide. **(B)**. Oxidized PcActx peptide. (ⅰ) Survival rates of zebrafish larvae. Data were expressed as means ± SD (n = 3). (ⅱ). Representative patterns of locomotion behavior of zebrafish larvae. The swimming trajectory was recorded every 10 min and is represented by curves. The instantaneous velocity was detected and displayed in different colors (black, <2 mm/s; green, 2–8 mm/s; red, >8 mm/s). (ⅲ). Changes in total distance travelled by period. (ⅳ). Statistical analysis of total distance travelled. Data are expressed as means ± SD (n = 10–12).

Survival rates of zebrafish larvae after 24 h exposure to PcActx peptides are shown in Figure 5. For linear PcActx peptide [Figure 5A (i)], the survival rate was maintained at about 100% when the animals were exposed to a concentration of 20 μM. However, treatment with 40 μM linear PcActx peptide for 24 h resulted in a reduction of survival rate to about 20%. For the oxidized PcActx peptide [Figure 5B (i)], survival decreased to low rates with concentrations lower than linear peptide, exhibiting a LC_50_ value of 22.8 μM compared to 31.5 μM. None of the zebrafish survived at the dosages of 40 μM or higher. In addition, the MTD values of linear and oxidized PcActx peptides were estimated as about 20 and 10 μM, respectively.

### PcActx Peptides Did Not Interfere With the Normal Locomotor Behavior of Zebrafish Larvae

Locomotion tests were performed to investigate the behavioral regulating effect on zebrafish larvae after 24 h exposure to linear and oxidized forms of PcActx peptide, only at the indicated concentrations below their MTD. Treatment with linear PcActx peptide at dosages ranging from 5 to 20 μM could not significantly regulate the total distance travelled by zebrafish larvae [Figure 5A (ii-iv)]. Similar results were observed when zebrafish larvae were treated with 2.5– 10 μM of oxidized PcActx peptide [Figure 5B (ii-iv)]. These results demonstrated that neither the linear nor oxidized PcActx peptides interfered with the locomotor behavior of zebrafish larvae at the various indicated concentrations. Concentrations that did not cause death or obvious behavioral deficits in zebrafish larvae were selected for assessing the protective effects against PTZ-induced epileptic seizure.

### PcActx Peptides Alleviated PTZ-Induced Hyperactivity in Zebrafish Larvae

**FIGURE 6:**
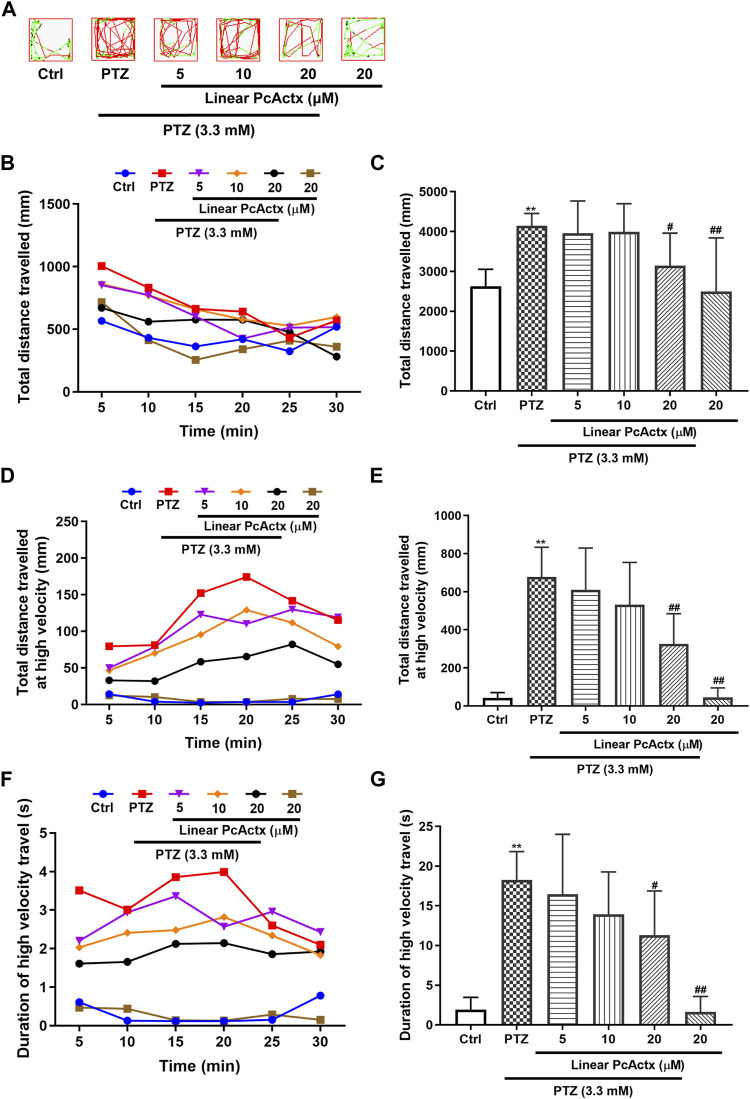
Linear PcActx peptide alleviated PTZ-induced seizure-related behavior in zebrafish larvae. **(A)** Representative patterns of behavioral locomotion in zebrafish larvae. The swimming trajectory was recorded every 5 min and is represented by curves. The instantaneous velocity was detected and displayed in different colors (black, <2 mm/s; green, 2–20 mm/s; red, >20 mm/s). **(B)**. Changes in total distance travelled by period. **(C)**. Statistical analysis of total distance travelled. **(D)**. Changes in total distance travelled at high velocity by period (>20 cm/s). **(E)**. Statistical analysis of total distance travelled at high velocity (>20 cm/s). **(F)**. Changes in the duration of high velocity travel by period (>20 cm/s). **(G)**. Statistical analysis of the duration of high velocity travel (>20 cm/s). Data are expressed as means ± SD (n = 9–10). * *p* < 0.05 vs Ctrl group, ** *p* < 0.01 vs Ctrl group, # *p* < 0.05 vs PTZ group, ## *p* < 0.01 vs PTZ group.

**FIGURE 7:**
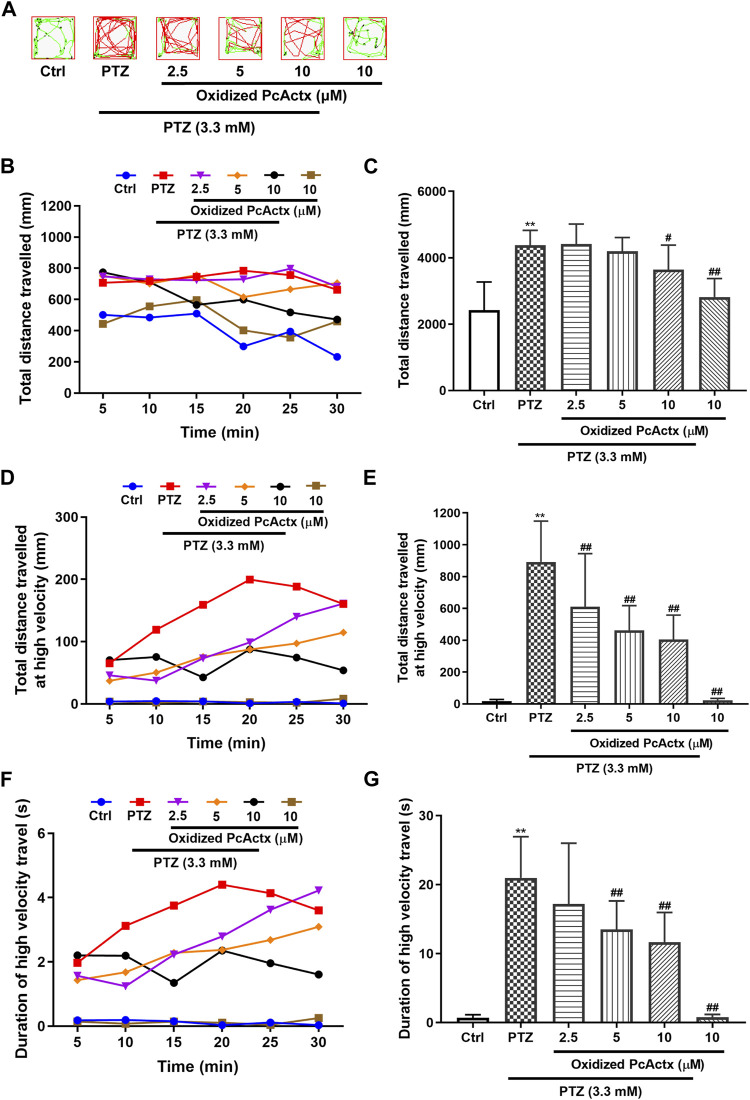
Oxidized PcActx peptide inhibited PTZ-induced hyperactivity in zebrafish larvae. **(A)** Representative patterns of locomotion behavior in zebrafish larvae. The swimming trajectory was recorded every 5 min and is represented by curves. The instantaneous velocity was detected and displayed in different colors (black, <2 mm/s; green, 2–20 mm/s; red, >20 mm/s). **(B)**. Changes in total distance travelled by period. **(C)**. Statistical analysis of total distance travelled. **(D)**. Changes in total distance travelled at high velocity by period (>20 cm/s). **(E)**. Statistical analysis of total distance travelled at high velocity (>20 cm/s). **(F)**. Changes in duration of high velocity travel by period (> 20 cm/s). **(G)**. Statistical analysis of the duration of high velocity travel (>20 cm/s). Data are expressed as means ± SD (n = 9–10). * *p* < 0.05 vs Ctrl group, ** *p* < 0.01 vs Ctrl group, # *p* < 0.05 vs PTZ group, ## *p* < 0.01 vs PTZ group.

PTZ, a non-competitive GABA antagonist, has been widely used to induce seizures for studying the effectiveness of novel antiepileptic drugs (AEDs) (Baraban et al., 2005; Ellis et al., 2012). PTZ-induced seizures elicited behavioral change, such as clonic-like convulsions (Baraban et al., 2005). As shown in Figures 6, 7, 3.3 mM PTZ induced notable hyperactivity in zebrafish larvae, which clearly elevated the total distance travelled and total distance travelled at high velocity (>20 mm/s), and also prolonged the duration of high velocity travel. However, PTZ-induced seizure-related behaviors in zebrafish were efficaciously prevented by PcActx peptides. Linear PcActx peptide clearly decreased the total swimming distance at a concentration of 20 μM (Figures 6B,C). Obviously, the total distance travelled at high velocity was inhibited by pretreatment with 20 μM linear PcActx peptide (Figures 6D,E). Similarly, a reduction in the total duration of high velocity travel was also observed in the treatment group with 20 μM linear PcActx peptide (Figures 6F,G). The oxidized PcActx peptide exhibited a more pronounced effect than the linear version in terms of maintaining normal locomotor behavior (Figure 7). Oxidized PcActx peptide significantly decreased the total distance travelled at a dosage of 10 μM (Figures 7B,C), and dramatically reduced the total distance travelled at high velocity when the concentration was higher than 1.25 μM (Figures 7D,E). Quantitative analysis of the duration of high velocity travel also supported a protective effect of oxidized PcActx peptide on locomotor behavior (Figures 7F,G).

### PcActx Peptides Prevented PTZ-Induced Up-Regulation of *c-fos* and *npas4a*

**FIGURE 8:**
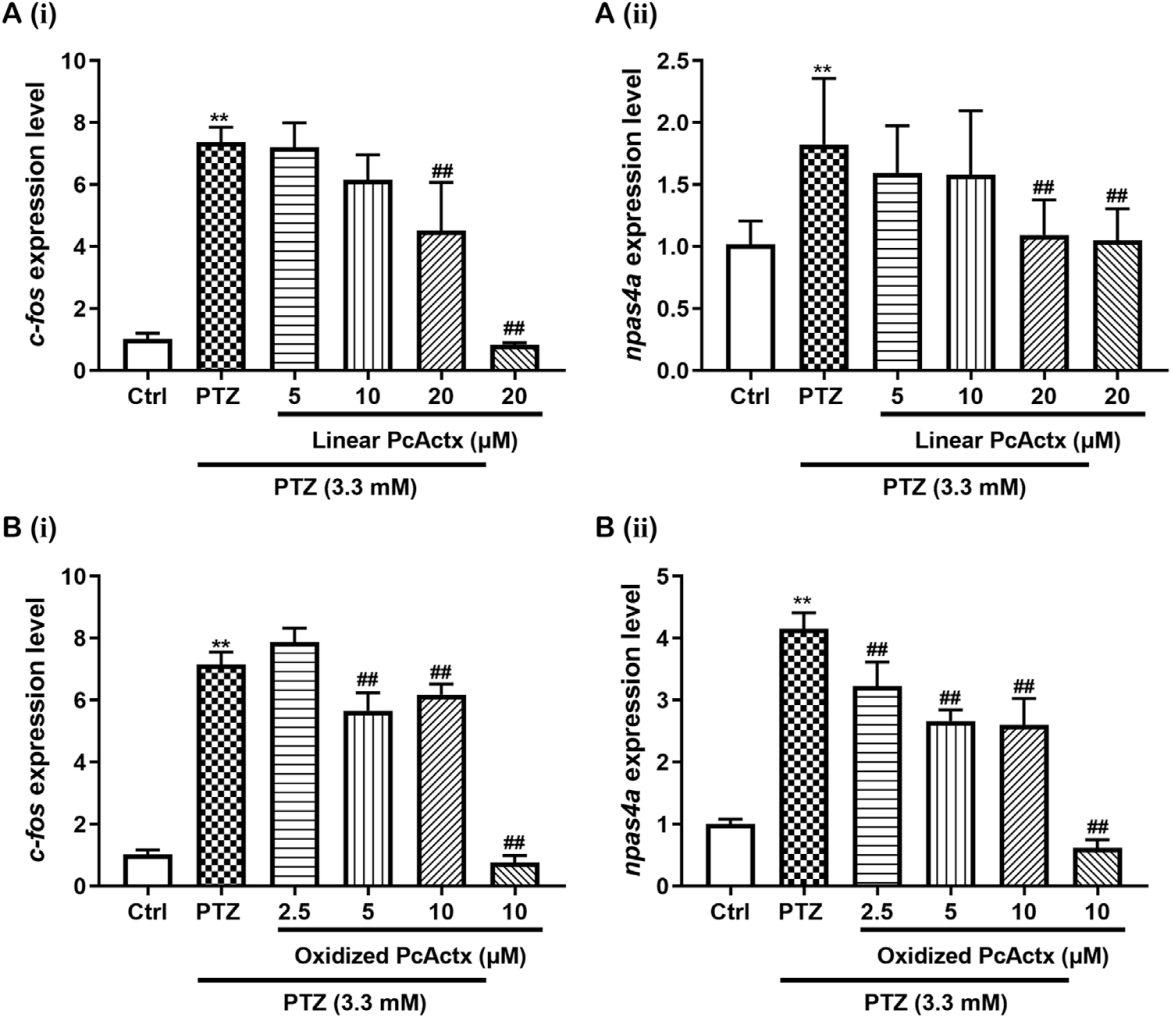
PcActx peptides prevented IER gene expression in PTZ-induced seizures of zebrafish. **(A)** Linear PcActx peptide. **(B)**. Oxidized PcActx peptide. (ⅰ). *c-fos* expression level. (ⅱ). *npas4a* expression level. Data are expressed as means ± SD (*n* = 4–6). * *p* < 0.05 vs Ctrl group, ** *p* < 0.01 vs Ctrl group, # *p* < 0.05 vs PTZ group, ## *p* < 0.01 vs PTZ group.

In addition to behavioral change, exposure to PTZ could result in an alteration in the expression of immediate early response (IER) genes. Both *c-fos* and *npas4a* are synaptic-activity-regulated genes that are known to be up-regulated by PTZ treatment (Baxendale et al., 2012; Torres-Hernandez et al., 2016). To further confirm the protective effect of PcActx peptides against PTZ-induced epileptic seizure, mRNA expression levels of *c-fos* and *npas4a* were determined by RT-PCR. As depicted in Figure 8, significant up-regulation of *c-fos* and *npas4a* was observed in the PTZ-treated groups, as compared to the control groups. Linear PcActx peptide was not able to suppress *c-fos* or *npas4a* expression until the concentration reached 20 μM (Figure 8A). Strikingly, both 5 and 10 μM oxidized PcActx peptide noticeably inhibited *c-fos* and *npas4a* expression (Figure 8B). Moreover, in the absence of PTZ treatment, the peptides did not induce significant changes in *c-fos* or *npas4a* expression, in parallel to the control groups.

### PcActx Peptides Could Inhibit PTZ-Induced ROS Accumulation in Zebrafish

**FIGURE 9:**
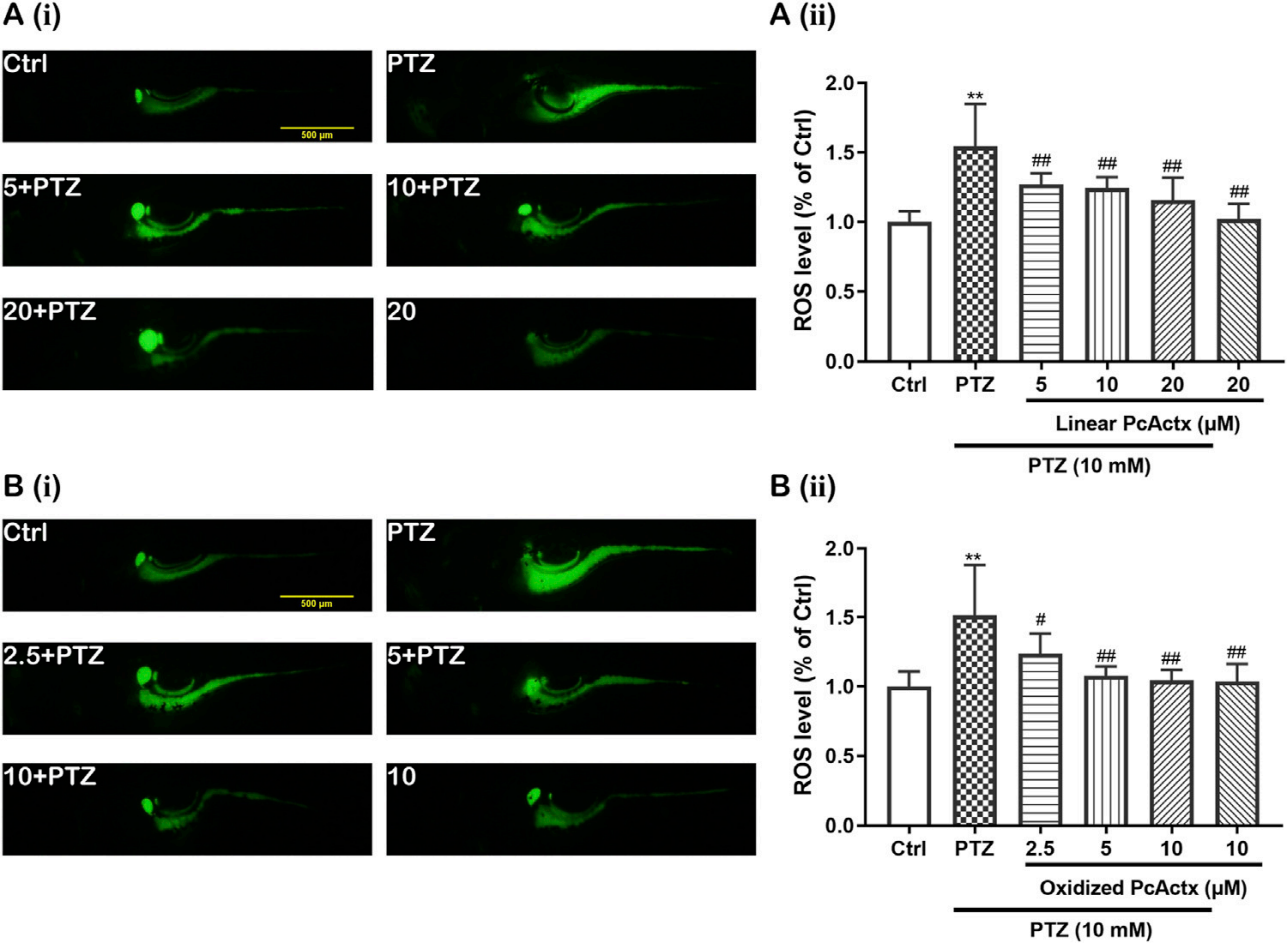
PcActx peptides attenuated PTZ-induced accumulation of ROS in zebrafish larvae. **(A)**. Linear PcActx peptide. **(B)**. Oxidized PcActx peptide. (i). Representative micrographs of fluorescence. (ii). Statistical analysis of ROS production. Data are expressed as means ± SD (n = 6–8). * *p* < 0.05 vs Ctrl group, ** *p* < 0.01 vs Ctrl group, # *p* < 0.05 vs PTZ group, ## *p* < 0.01 vs PTZ group.

It is well known that oxidative stress can be a cause and consequence of epileptic seizures (Patel, 2004). Mitochondrial oxidative stress and dysfunction might contribute to seizure-related brain damage and can also render the brain more susceptible to epileptic seizures (Patel, 2004). As displayed in Figure 9, PTZ obviously increased the ROS level in zebrafish larvae. However, the excessive production of ROS triggered by PTZ was effectively decreased by linear PcActx peptide (Figure 9A). Moreover, oxidized PcActx peptide also dose-dependently attenuated PTZ-induced ROS accumulation in zebrafish larvae, especially at 5 and 10 μM (Figure 9B). Meanwhile, application of PcActx peptides alone, either in linear or oxidized form, did not induce abnormal generation of ROS when compared to the control group. The results regarding locomotion behavior, mRNA levels of *c-fos* and *npas4a*, and ROS production (Supplementary Table S2) all indicated that the protective activity of oxidized PcActx peptide was more potent than that of the linear form.

### Oxidized PcActx Peptide Reduced the Expression Level of Several Genes Related to Calcium and GABAergic-Glutamatergic Signaling

**FIGURE 10:**
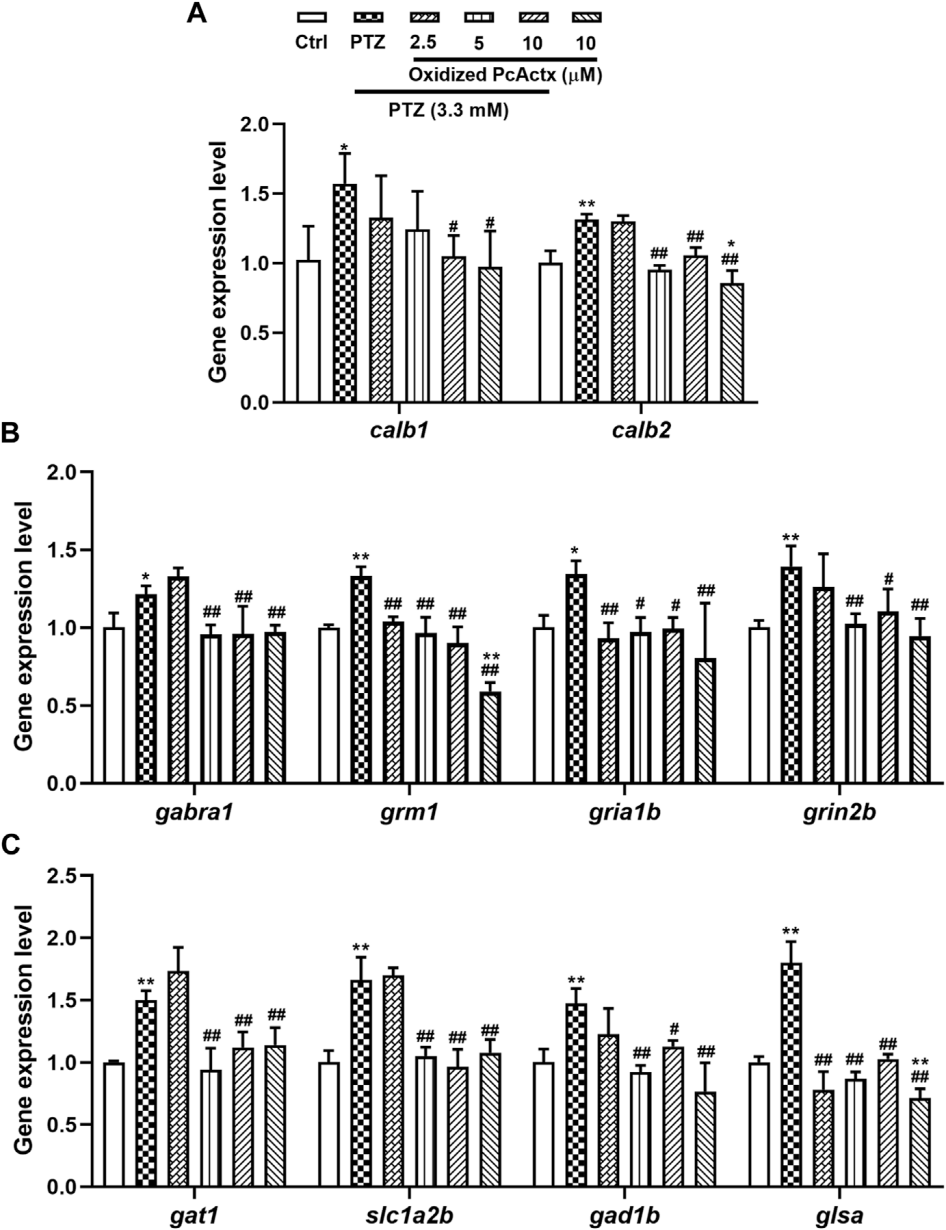
Oxidized PcActx peptide could regulate calcium and glutamatergic-GABAergic signaling related genes in PTZ-stimulated epileptic zebrafish. **(A)** *calb1* and *calb2* expression levels. **(B)**. *gabra1, grm1, gria1b* and *grin2b* expression levels. **(C)**. *gat1, slc1a2b, gad1b* and *glsa* expression levels. Data are expressed as means ± SD (*n* = 4). * *p* < 0.05 vs Ctrl group, ** *p* < 0.01 vs Ctrl group, # *p* < 0.05 vs PTZ group, ## *p* < 0.01 vs PTZ group.

In comparison with the control group (Figure 10), the PTZ-treated group showed a remarkable elevation in the mRNA expression of genes related to calcium signaling and glutamate-GABA metabolic signaling, including *calb1*, *calb2, gabra1, grm1, gria1b, grin1b, gat1, slc1a2b, gad1b,* and *glsa*. In contrast, oxidized PcActx peptide conspicuously modulated the abnormal expression levels of *calb1*, *calb2, gabra1, grm1, gria1b, grin1b, gat1, slc1a2b, gad1b,* and *glsa.* Furthermore, treatment with oxidized PcActx peptides alone (10 μM) also slightly reduced the expression of *calb2, grm1, gria1b, gad1b,* and *glsa*, even though no significant differences were observed in *gria1b, gad1b,* and *glsa* when compared to control group. These data provided clear evidence that the anti-epileptic activity of PcActx peptide may involve modulation of calcium signaling and GABAergic-glutamatergic signaling.

## Discussion

Improvements of transcriptomics and proteomics techniques, and widespread application thereof, led to significant advances in animal venom-peptide discovery. Several venom-derived drugs have been developed and approved for treatment of various diseases, owing to their extremely high specificity and potency for particular molecular targets (King, 2011; Robinson et al., 2017). For instance, ziconotide (Prialt^®^), a blocker of the Cav2.2 channel, is used to treat chronic pain. In addition, a number of animal venom-like proteins and peptides are currently in clinical trials (ShK-192, α-cobrotoxin) and preclinical studies (APETx2, Vicrostatin) (King, 2011; Robinson et al., 2017). Venom-peptides from cnidarians, especially sea anemones, have attracted growing interest in the context of the discovery and development of drug therapies, due to their potential for targeting various ion channels (Mouhat et al., 2004). However, proteinaceous (proteins and peptides) toxins originating from other species of Cnidaria, like the zoantharians, are largely underexploited. In the present study, we identified a novel neuropeptide through transcriptomics analysis of zoantharian *P. caribaeorum*, and investigated its anti-epileptic potential and underlying mechanism of action using a PTZ-induced seizure model of zebrafish larvae.

A novel peptide, named PcActx, was identified on the transcriptome of *P. caribaeorum*. Based on the maximum likelihood tree (Figure 1B), PcAcxt peptide was well clustered based on APHC and HCRG21 peptide phylogeny, where those peptides are TRPV1 channel inhibitors from the sea anemone *H. crispa*. The data obtained through molecular phylogenetic analysis and structural alignment showed that PcActx peptide shared a similar domain with APHC peptides (Figures 1A, 2E). Unlike APHC peptides, PcActx peptide contains three cysteine residues and folds only one disulfide bond with one free cysteine residue. PcActx peptide functions as a monomer rather than a dimer, which might be due to the unique spatial structure of Cys 19 (Figures 2A,B, 3E). A disulfide bridge forming between thiol groups in two cysteine residues is an important component of the secondary and tertiary structure of proteins and polypeptides for maintaining their structural stability and function (Thornton, 1981). *In silico* studies proved that cysteine-rich peptides, such as MCoTI-II and μ-Conotoxin PIIIA, showed different dynamic characteristics in terms of disulfide connectivity (Tietze et al., 2012; Zhang et al., 2016). *In vitro* and *in vivo* studies demonstrated that different disulfide bridge patterns in the same sequence resulted in different bioactivities. Tietze et al. (2012) reported that the ability of μ-Conotoxin PIIIA to block Na_V_1.4-mediated ion currents varied depending on disulfide connectivity. Moreover, the unoxidized isomer cannot adopt an adequate conformation to block the Na_V_ channel. Meanwhile, the ribbon disulfide isomer of α-Conotoxin AuIB, a non-native structure, has approximately 10 times greater potency to block nicotinic acetylcholine receptors than the native peptide (Dutton et al., 2002). In addition, our previous study found that oxidized PpVα peptide with a single disulfide bridge was more efficacious than the linear form in epileptic and neurodegenerative models (Liao et al., 2019). Although the single free cysteine in the sequence might partly increase instability, other previous study also observed a peptide with a similar distinctive cysteine spacing pattern that remained biologically active; for example, Rattusin, an intestinal α-defensin-related peptide, contained five cysteine residues and exhibited salt-insensitive antibacterial properties (Patil et al., 2013). In this study, the anti-epileptic activities of linear and single disulfide bond oxidized PcActx peptides, which share the same primary amino acid sequence, were determined and compared.

Since PcActx peptide is homologous to APHC peptides that act on the TRPV1 channel, an interaction of PcActx peptide with the TRPV1 channel was expected. The peptide-protein docking analysis indicated that PcActx peptide has the potential to interact with the TRPV1 channel via four hydrogen bonds in pre-S1 helix, S4-S5 linker and TRP domain (Figures 4C,D). Furthermore, Fluorescent calcium measurement displayed that PcActx peptides conspicuously inhibited the capsaicin-induced calcium response via the TRPV1 channel inactivation (Figures 4E,F). The TRP domain, a unique structural feature of TRP channels, selectively interacts with the pre-S1 helix and S4-S5 linker via salt bridging and hydrogen bonding, which allosterically affects pore conformation (Liao et al., 2013). Moreover, cation-π interactions have been observed between the S4-S5 linker and S5 helix, thus achieving functional coupling (Liao et al., 2013). Covalent intra-subunit interactions have also been observed between the S4-S5 linker and S5-P-S6 pore region (Liao et al., 2013). Hence, interactions with these structures might affect TRPV1 channel activation.

In addition to its widely validated role in pain signal transduction, TRPV1 channel activation may also contribute to epileptogenesis. In an evaluation of the neuroprotective effect of PcActx peptides, PTZ, an anxiogenic epileptogenic compound, was widely utilized to induce an epileptic response in animal models. Zebrafish emerges as a successful experimental model for neurological disease due to its remarkable features, including low maintenance cost, high fecundity, rapid development and transparence (Hortopan et al., 2010; Barbalho et al., 2016). Due to the small size of zebrafish larvae, their behaviors could be further analyzed using a locomotion tracking system for the quantification of movement (Baraban et al., 2005). More importantly, zebrafish genes share approximately 70–80% homology to the human genome (Hortopan et al., 2010; Barbalho et al., 2016).

In the present study, PTZ provoked extremely abnormal behavioral changes (Figures 6, 7) and IER gene overexpression (*c-fos* and *npas4a*, Figure 8) in zebrafish larvae. Both *c-fos* and *npas4a* are synaptic-activity-regulated genes, which can be up-regulated by PTZ stimulation (Baxendale et al., 2012; Torres-Hernandez et al., 2016). *c-fos* acts as the gold standard for measuring synaptic function in the central nervous system (CNS), which can be induce transiently in response to seizure onset (Baraban et al., 2005; Baxendale et al., 2012; Torres-Hernandez et al., 2016). The transcription factor *npas4a* regulate the development of inhibitory synapses through controlling the expression of a set of activity-dependent genes, thus maintaining the balance between excitatory and inhibitory synapses within neural circuits (Lin et al., 2008; Spiegel et al., 2014). Pre-treatment with PcActx peptides (both linear and oxidized isomers) significantly alleviated PTZ-induced seizure-related behaviors, including the total distance travelled, total distance travelled at high velocity (>20 mm/s) and duration of swimming at high velocity (Figures 6, 7). Overexpression of *c-fos* and *npas4a* stimulated by PTZ was dramatically reduced by PcActx peptide treatment (Figure 8), suggesting that the PcActx peptides inhibited IER gene expression, thereby regulating the balance of synaptic excitatory and inhibitory activities.

ROS generation plays a critical role in epileptogenesis, serving as both contributor to and consequence of epilepsy (Patel, 2004). Similarly, ROS can be induced by activation of the TRPV1 channel via Ca^2+^ influx, and also mediates TRPV1 activation (Naziroglu, 2015). Our investigation showed obviously abnormal ROS production in PTZ-treated groups. However, PcActx peptides, as potential TRPV1 modulators, considerably decreased ROS accumulation (Figure 9), which manifested in PcActx peptide exerting favorable antioxidative properties against pathologic alterations induced by PTZ stimulation. Data regarding the anti-epileptic effects of linear and oxidized PcActx peptides are summarized in Supplementary Table S2. We found that both linear and oxidized PcActx peptides could notably alleviate PTZ-induced epileptic seizure in zebrafish larvae, but the single disulfide oxidized PcActx peptide was more effective than the linear one. Our previous study also found that the oxidized form of PpVα peptide exhibited higher bioactivity than its linear counterpart, probably due to higher structural stability and binding affinity to the target receptor (Liao et al., 2019). Therefore, the oxidized PcActx peptide was subjected to further examination with respect to the upstream signaling pathways underlying its anti-epileptic activity.

Gene *calb1* and *calb2* encode intracellular calcium-binding proteins, which are called calbindin 1 and calbindin 2 (also known as calretinin) (Parmentier et al., 1991). The calbindins are involved in several cellular functions, such as signaling transduction and calcium homeostasis (Xu and Tang, 2018). Calretinin, expressed by GABAergic interneurons in the hippocampus, also functions as a modulator of neuronal excitability, which can regulate synaptic plasticity and transmission (Camp and Wijesinghe, 2009). The sensitivity of calretinin-positive interneurons is responsible for the impaired dendritic inhibition seen in epilepsy (Toth and Magloczky, 2014). The abnormal expression of calbindin-1 positive nerve cells might also participate in the process of epileptogenesis (Thom et al., 2000; Wittner et al., 2002). In the CNS, glutamate and GABA are the most important excitatory and inhibitory neurotransmitters, respectively, and are responsible for mediating the neuronal activity. Several proteins and/or genes are necessary in glutamate-GABA metabolic pathways and may be altered in epilepsy. Fast-inhibitory postsynaptic potential can be induced by the activation of GABA_A_ receptor α 1 (Gabra1), which belongs to the GABA_A_ receptor family (Kuriyama et al., 1993; Najm et al., 2001). Gene *grm1* encodes a metabotropic glutamate receptor (mGluR), which is a second-messenger coupled receptor involved in neuronal plasticity and epilepsy (Najm et al., 2001). N-methyl-D-aspartate (NMDA) receptors (encoded by the *grin* gene) and α-amino-3-hydroxy-5-methyl-4-isoxazolepropioniac acid (AMPA) receptors (encoded by the *gria* gene) are ionotropic glutamate receptors (iGluR), the activation of which leads to glutamate accumulation, overexcitement of synapses, and, ultimately, epilepsy (Najm et al., 2001; Rogawski and Loscher, 2004). GABA transporter 1 (GAT 1) is present in the cell membrane of neurons and astrocytes, and its primary function is to remove or reuptake GABA from the synaptic cleft, thereby inhibiting or terminating the synaptic actions of GABA (Meldrum and Rogawski, 2007; Scimemi, 2014). Excitatory amino acid transporter 2 (EAAT2) is encoded by the *slc1a2* gene and is responsible for over 90% of the glutamate reuptake in the forebrain (Holmseth et al., 2009), which is implicated in the regulation of synaptic activity and plasticity (Takahashi et al., 2015). Glutamate decarboxylase 1 (GAD1), encoded by *gad1*, is an enzyme that catalyzes the decarboxylation of glutamate to GABA, thus regulating the steady-state GABA concentration (Petroff, 2002). Glutaminase (encoded by the gene *glsa*) participates in the glutamate-glutamine cycle, where glutamate is formed from phosphate-activated glutaminase (Hertz and Zielke, 2004). In the present study, treatment with the oxidized PcActx peptide dramatically reduced the overexpression of *calb1, calb2, gabra1, grm1, gria1b, grin1b, gat1, slc1a2b, gad1b, and glsa*, the levels of which were increased by PTZ (Figure 10). Taken together, these results indicated that PcActx peptide exhibits prominent anti-epileptic activity, potentially involving modulation of calcium signaling and GABAergic-glutamatergic signaling.

**FIGURE 11:**
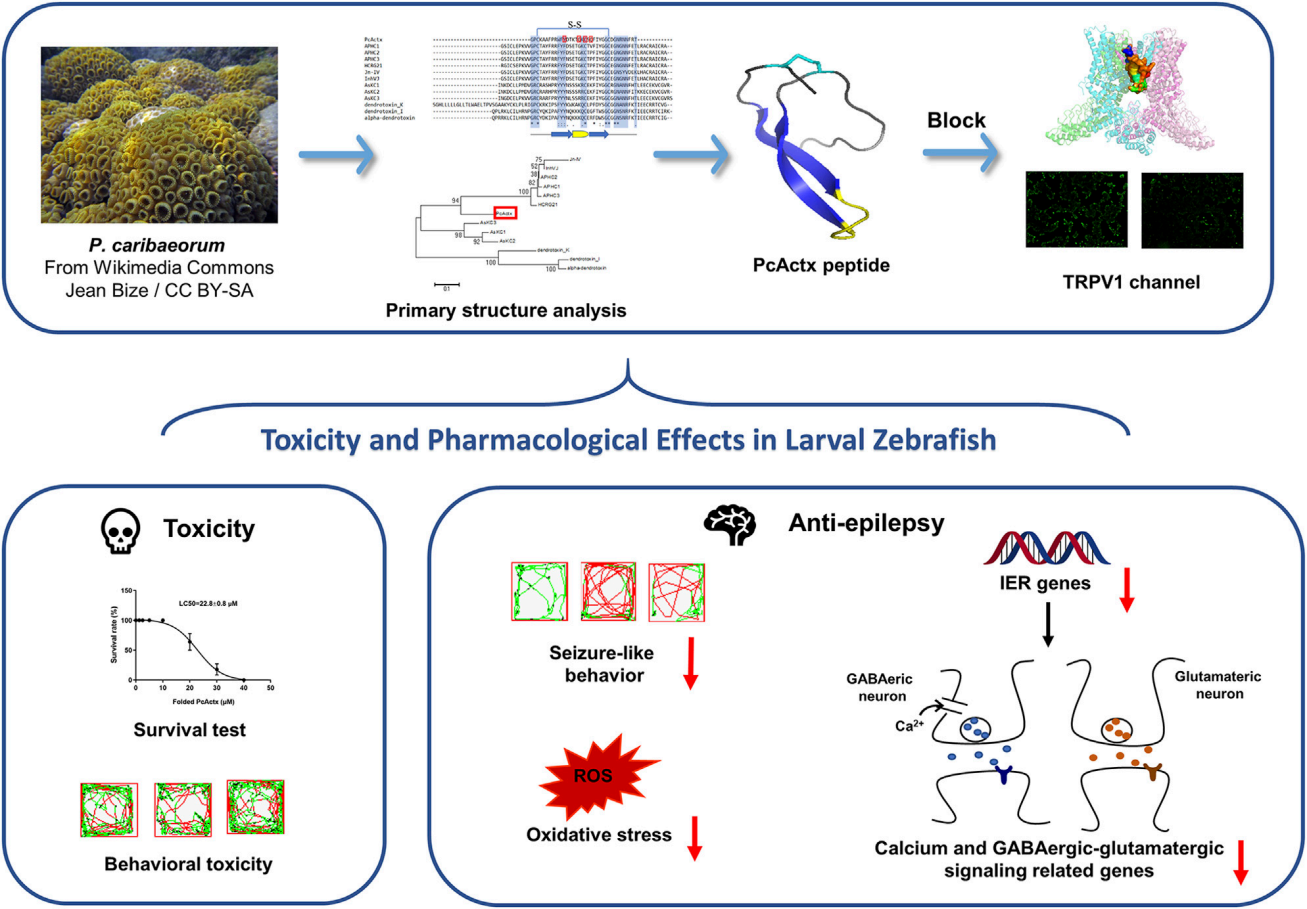
A schematic diagram. PcActx peptide from transcriptome of zoantharian *P. caribaeorum* exhibits prominent anti-epileptic activity, probably through modulating calcium signaling and GABAergic-glutamatergic signaling.

In conclusion, PcActx peptide was characterized as a novel APHC homologue that seems to act on the TRPV1 channel. The bioactivity validation results comprehensively demonstrated that both linear and oxidized PcActx peptides not only effectively prevented PTZ-induced seizure-related behaviors, but also efficaciously suppressed the overexpression of *c-fos* and *npas4a*, as well as excessive ROS production, which are known biomarkers of neuronal injury. In particular, oxidized PcActx peptide clearly inhibited the mRNA expression levels of *calb1, calb2, gabra1, grm1, gria1b, grin2b gat1, slc1a2b, gad1b,* and *glsa*. Thus, PcActx peptide represents a promising therapeutic candidate for epilepsy management, the mechanism of action of which may involve regulation of calcium and GABAergic-glutamatergic signal pathways (Figure 11).

## Data Availability

The original contributions presented in the study are included in the article/Supplementary Material, further inquiries can be directed to the corresponding authors.

## Abbreviations

AEDs, antiepileptic drugs; AMPA, α-amino-3-hydroxy-5-methyl-4-isoxazolepropioniac acid; APHC, analgesic polypeptides from sea anemone Heteractis crispa; ASIC, acid-sensing ion channel; CaV, voltage-gated calcium channel; CNS, central nervous system; dpf, day post-fertilization; EAAT2, Excitatory amino acid transporter 2; GABA, γ-aminobutyric acid; Gabra1, GABAA receptor α 1; GAD1, glutamate decarboxylase 1; GAT 1, GABA transporter 1; GAT 1, GABA transporter 1; IER, immediate early response; iGluR, ionotropic glutamate receptors; KV, voltage-gated potassium channel; MD, molecular dynamics; mGluR, metabotropic glutamate receptor; MTD, maximum tolerated doses; MW, molecular weight; NaV, voltage-gated sodium channel; NMDA, N-methyl-D-aspartate; PTZ, pentylenetetrazol; RMSD, root-mean squared deviation; SCG, superior cervical ganglion; ShK, Stichodactyla toxin; TRP, transient receptor potential cation channel; TRPV1, transient receptor potential cation channel subfamily V member 1.
