# Supplementary material for: Toxic Peptide From Palythoa caribaeorum Acting on the TRPV1 Channel Prevents Pentylenetetrazol-Induced Epilepsy in Zebrafish Larvae
Source: Front Pharmacol. 2021 Dec 1;12:763089. doi: 10.3389/fphar.2021.763089 (PMC8672801; doi:10.3389/fphar.2021.763089)
Supplement: Supplementary file 1 [file DataSheet1.docx]

Supplementary Material

**Supplementary Table 1** Primers for RT- PCR

| Gene | Primer sequence 5’-3’ |
| --- | --- |
| *ef1a* | Forward - GCTCAAACATGGGCTGGTTC |
|  | Reverse - AGGGCATCAAGAAGAGTAGTACCG |
| *c-fos* | Forward - TTACCCGCTCAACCAGACTC |
|  | Reverse - TGACAGTTGGCACGAAAGAG |
| *npas4a* | Forward - GAGTAACCTGGTGCCTCCAA |
|  | Reverse - TTTGCCTACGCACTGATTTG |
| *calb1* | Forward - GCATGTTTGTAGCTGACGGC |
|  | Reverse - ATAGAGACAGGACTGGGGCG |
| *calb2* | Forward - ACGACAAGGATGGCAATGGT |
|  | Reverse - AGCGCCATGATGCTCTTCTT |
| *gabra1* | Forward - TCAGGCAGAGCTGGAAGGAT |
|  | Reverse - TGCCGTTGTGGAAGAACGT |
| *grm1* | Forward - GGCTGGGTTAGGAAGTGCAT |
|  | Reverse - TGATTTTCAACCTGGCCCCT |
| *gria1b* | Forward - TGTCCACAAATCCCTCCGAAT |
|  | Reverse - ATTCGCCATGGGTATGCCAG |
| *grin2b* | Forward - TACGGGTACACCTGGATCGT |
|  | Reverse - TCATCATGGTGGAGGTTGCC |
| *gat1* | Forward - ATGCTGTTTATCCTGTTCATCCG |
|  | Reverse - TGTTGAAGGGGTTGTAGCTCC |
| *slc1a2b* | Forward - CAGTGCGTCTTTGCTGCTCAA |
|  | Reverse - TTGGCCTCACATTCTCGTGT |
| *gad1b* | Forward - AACTCAGGCGATTGTTGCAT |
|  | Reverse - TGAGGACATTTCCAGCCTTC |
| *glsa* | Forward - AGGCCATGCTGAGGTTG |
|  | Reverse - CTGCCGTCTCTTTTTCGCT |

**Supplementary Table 2** Comparisons on protective effects of linear and oxidized PcActx peptides in PTZ-induced epilepsy

| Peptide | LC_50_ (μM) | MTD  (μM) | Dosage (μM) | Restoration as compared to PTZ | | | | | |
| --- | --- | --- | --- | --- | --- | --- | --- | --- | --- |
|  |  |  |  | Total distance  (%) | Total distance in high velocity (%) | Total duration in high velocity (%) | *c-fos*  (%) | *npas4a*  (%) | ROS  (%) |
| Linear PcActx | 31.5±0.6 | 20 | 5 | 4.56 | 9.89 | 11.64 | 2.20 | 12.58 | 17.75* |
|  |  |  | 10 | 3.55 | 21.46 | 27.70 | 16.42 | 13.34 | 19.53* |
|  |  |  | 20 | 24.16* | 51.91* | 33.87* | 38.65* | 39.94* | 25.03* |
| Oxidized PcActx | 22.8±0.8 | 10 | 2.5 | -0.82 | 31.55* | 18.16 | -9.96 | 24.59 | 18.26* |
|  |  |  | 5 | 4.06 | 48.17* | 35.51* | 20.89* | 35.00* | 28.85* |
|  |  |  | 10 | 16.80* | 54.66* | 44.41* | 13.59* | 34.18* | 31.10* |

LC_50_ denoted the concentration of peptides that cause 50% of death in zebrafish larvae.

MTD denoted the highest concentration of peptides that did not cause obvious death in zebrafish larvae.

* indicated significant difference between treatment groups and PTZ groups

Restoration (%) = (Means of PTZ group – Means of treated groups) / Means of PTZ group


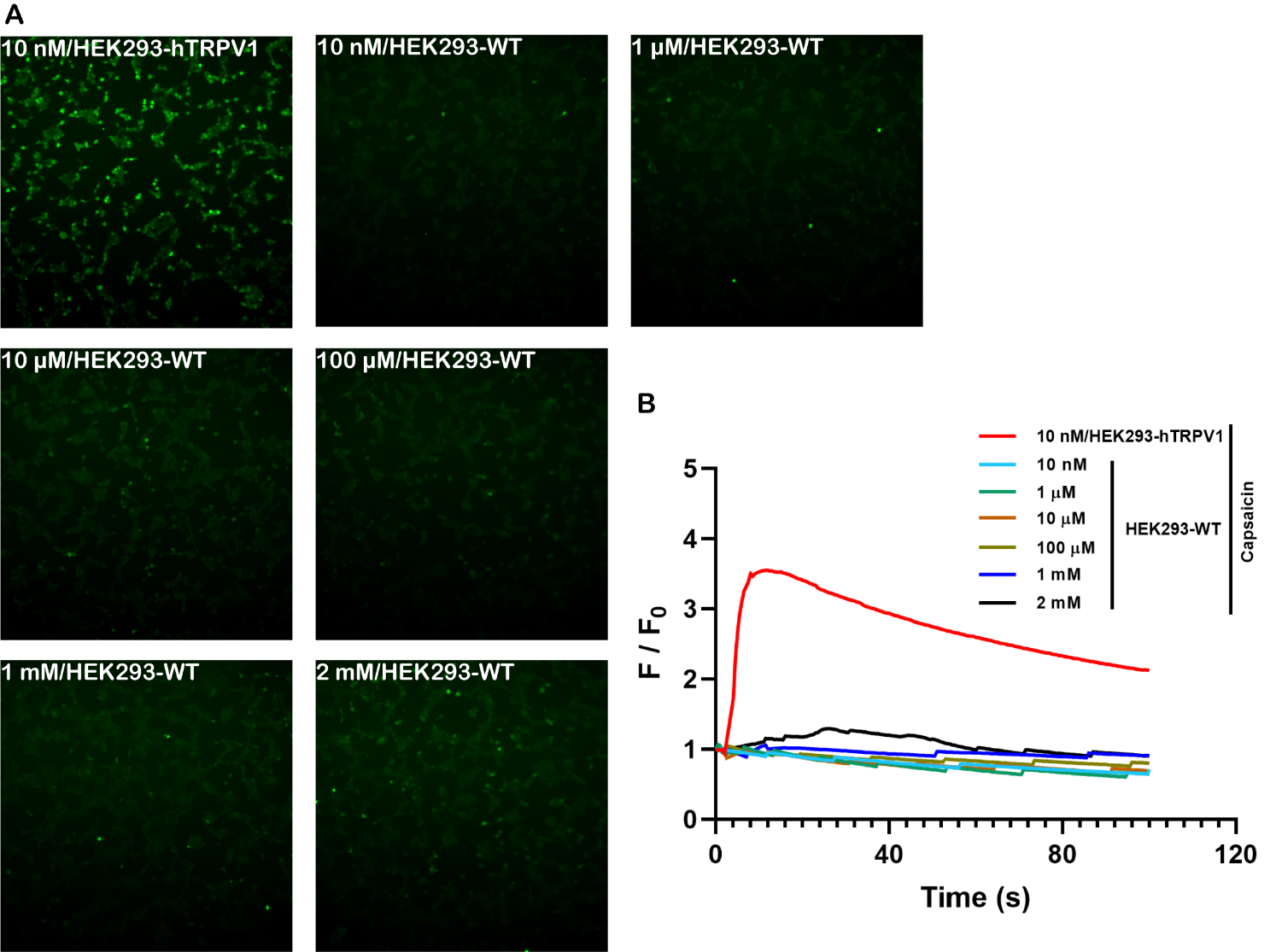


**Supplementary** **Figure 1** Capsaicin induced TRPV1 mediated calcium influx**. A.** Representative images of capsaicin-induced intracellular calcium concentration of HEK293-hTRPV1 cell and HEK293-WT cell (CAP: capsaicin; CPZ: capsazepine). F. Representative time-dependent response of Ca^2+^ fluorescence intensity in each group. Ca^2+^ responses were measured as changes in fluorescence intensity of the representative average plots (n = 5) before (F_0_) and after capsaicin addition (F).
